# Solid-State Nanopores for Spatially Resolved Chemical Neuromodulation

**DOI:** 10.1021/acs.nanolett.4c02604

**Published:** 2024-11-19

**Authors:** F. Vacca, F. Galluzzi, M. Blanco-Formoso, T. Gianiorio, A.F. De Fazio, F. Tantussi, S. Stürmer, W. Haq, E. Zrenner, A. Chaffiol, C. Joffrois, S. Picaud, F. Benfenati, F. De Angelis, E. Colombo

**Affiliations:** 1 Center for Synaptic Neuroscience and Technology, Istituto Italiano di Tecnologia, 16132 Genova, Italy; 2 IRCCS Ospedale Policlinico San Martino, 16132 Genova, Italy; 3 The Open University Affiliated Research Centre at Istituto Italiano di Tecnologia (ARC@IIT), 16132 Genova, Italy; 4 Plasmon Nanotechnology, Istituto Italiano di Tecnologia, 16163 Genova, Italy; 5 CINBIO Universidade de Vigo, 36310 Vigo, Spain; 6 Department of Neuroscience (DINOGMI), University of Genoa, 16132 Genova, Italy; 7 Centre for Ophthalmology, Institute for Ophthalmic Research, University of Tübingen, 72076 Tübingen, Germany; 8 Institut de la Vision, Sorbonne Université, 75012 Paris, France

**Keywords:** nanopores, glutamate stimulation, neuronal stimulation, drug delivery, neural interface

## Abstract

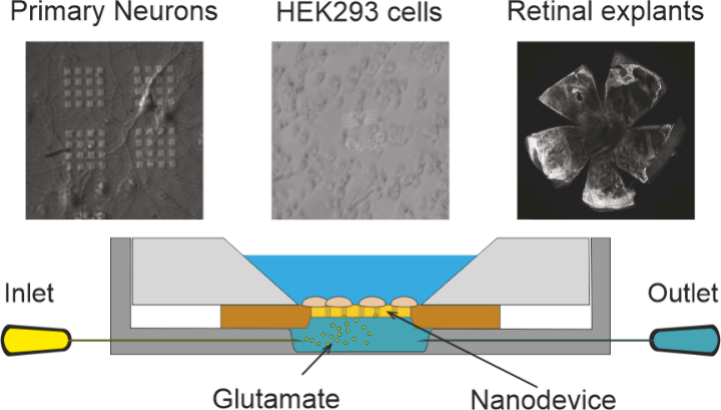

Most neural prosthetic devices are based on electrical stimulation, although the modulation of neuronal activity by a localized chemical delivery would better mimic physiological synaptic machinery. In the past decade, various drug delivery approaches attempted to emulate synaptic transmission, although they were hampered by poor retention of their cargo while reaching the target destination, low spatial resolution, and poor biocompatibility and stability of the materials involved. Here, we propose a planar solid-state device for multisite neurotransmitter translocation at the nanoscale consisting of a nanopatterned ceramic membrane connected to a reservoir designed to store neurotransmitters. We achieved diffusion-mediated glutamate stimulation of primary neurons, while we showed the feasibility to translocate other molecules through the pores by either pressure or diffusion, proving the versatility of the proposed technology. Finally, the system proved to be a promising neuronal stimulation interface in mice and nonhuman primates *ex vivo*, paving the way toward a biomimetic chemical stimulation in neural prosthetics and brain machine interfaces.

Neural communication relies on neurotransmitter release at axonal terminals, which, by triggering a change in the membrane potential of postsynaptic neurons, is responsible for information transfer to the downstream network. Nowadays, the most common way to replace a missing or dysfunctional presynaptic input is electrical stimulation of the denervated postsynaptic neuron. However, electrical stimulation produces a nonspecific depolarization of neuronal membranes affecting large volumes of cells and fibers *en passant*, hardly achieving the nanoscale of a synapse.

The nanoscaling of the microelectrode technology has shown potential for both neural stimulation and recording in few *in vitro* proof-of-concept studies,^1−3^ although the low current injection threshold imposed by smaller electrodes hampers an efficient stimulation. Likewise, nanoscale field-effect transistors achieved intracellular access with remarkable signal-to-noise ratios, but still lack the implementation of stimulation capabilities.^4,5^

The ability to mimic the chemical signaling of neurotransmission would represent a fully biomimetic approach for the therapy of a variety of neurological disorders, being able to exploit the innate specificity of endogenous membrane receptors.^6^

In the past decade, drug delivery technologies thoroughly investigated the release of molecules at the microscale either from tip electrodes or planar devices.^7^ Electrophoretic translocation of glutamate has been achieved by micropore arrays,^8^ while an iontronics-enhanced polymeric device showed a millisecond delivery of acetylcholine.^9^ Furthermore, a flexible solution incorporating a parylene microtube array demonstrated controlled delivery of fluorescent molecules with temporal and spatial precision.^10^

While these studies are limited to the proof-of-concept translocation of molecules and neurotransmitters, a few translations of the technology to biological models have been reported. Examples are represented by silicon nitride membranes featuring 10 μm apertures employed for a bradykinin-based stimulation of PC12 cells,^11^ or a porous commercial membrane able to permeate acetylcholine, successfully mediating the stimulation of TE671 cells expressing acetylcholine receptors.^12^

On the other hand, perforated multielectrode array technology has been declined in a variety of architectures including state-of-the-art materials such as graphene.^13^ A few reports describe alternative chemical-based stimulation of neurons at the microscale, exploiting simultaneous functional recordings. A polymeric 25 μm microchannel device, coupled to a fluidic valve system, exhibited precise control of glutamate stimulation of hippocampal neurons as depicted by simultaneous Ca^2+^ imaging,^14^ while an 8-output fluidic chip showcased selective and patterned glutamate release on retinal explants as revealed by multielectrode array recordings.^15^ Finally, silicon-based out-of-plane high-density microneedles with blunt or beveled tips have been proposed as *in vivo* neural interface with drug delivery potentiality.^16,17^

Despite offering a promising approach to mimic chemical signaling between cells, current microtechnological tools encounter scalability and resolution challenges, especially when applied to complex neuronal networks and associated fluidic systems.^6^ Very few nanofluidic strategies in association with neurons have been investigated thus far, although a spatially localized intracellular delivery of exogenous molecules was recently demonstrated on nanostructured microelectrode arrays by means of electroporation-assisted membrane permeabilization in primary neurons.^18^ Moreover, protruding 3D nanochannels revealed to be a versatile tools for on-demand delivery of molecules and particles exploiting either electroporation or plasmonic opto-poration on different models of adherent cells, from neurons to cardiomiocytes.^19−21^

Notwithstanding the potential of the planar intracellular technology, a tool capable of repeatedly releasing chemically active compounds in small volumes at the nanoscale, without the need for electrodes or plasmonic elements, would be a potent asset for a biomimetic neuronal stimulation in both clinical and laboratory settings. Here, we characterized a planar neural interface based on the diffusion-driven release of neurotransmitters from a solid-state array of nanopores for the development of a spatially resolved and biomimetic stimulation platform. The system was integrated with microfluidics to realize a multipurpose lab-on-chip for the spatially confined release of bioactive molecules,^22^ and can be easily coupled with nanoelectrodes and plasmonics, providing a further degree of versatility to the nanodevices together with a higher spatial resolution.^23,24^

We achieved diffusion-mediated glutamate stimulation of primary neurons cultured onto the planar solid-state device as well as of inner neurons from rat and macaque retinal explants. The system, capable of translocating molecules through the pores by either pressure or diffusion, appears to be an efficient platform for chemical stimulation of neural prosthetics at the nanoscale.

We realized a nanopore array on a suspended Si_3_N_4_ membrane out of a silicon frame capable of passively releasing small volumes of neurotransmitter at the nanoscale (Figure 1a, left). The device is realized on a commercial silicon wafer covered by 500 nm of Si_3_N_4_ on both sides. Conventional UV photolithography was employed to fabricate the suspended membrane over an area ranging from 0.4 × 0.4 to 2.5 × 2.5 mm^2^, according to the experimental requirements for adherent cells or retinal explants (Figure 1b and Figure S1a). A thin film of Ti and Au was then deposited on the membrane to allow for Focused Ion Beam milling (FIB) of nanopore arrays deputed to the localized neurotransmitter-based stimulation of neuronal networks (Figure 1a, right). The nanopore arrays designed for the various experiments are shown in Figure S1b. Top view and cross section of a single nanopore show a diameter of ≈100 nm (Figure 1c and Figure S1c). The device was encased in a customized microfluidic system enabling the positioning of the device between two watertight chambers separated only by the nanopores (Figure 1d). The time-dependent diffusive translocation of glutamate from a reservoir (10 mM concentration) through the nanodevice, investigated by using a bioluminescence glutamate assay, demonstrated an efficient and reproducible effect over different devices (Figure 1e). These results are corroborated by previous reports in which the authors show through a finite elements simulation the pure diffusion of molecules through similar nanostructures. In the previous study, the delivery rate of propidium iodide from slightly smaller pores (90 nm) was estimated 10^5^ molecules per second per pore for a concentration of 1.5 μM and an injection time of 5 min.^23^ Considering our reservoir concentration of 10 mM, the number of molecules increases approximately to 6 × 10^5^ against 2 × 10^6^ molecules per second per pore achieved by the experiment of Figure 1e, the difference being ascribable to the different experimental conditions.

**Figure 1:**
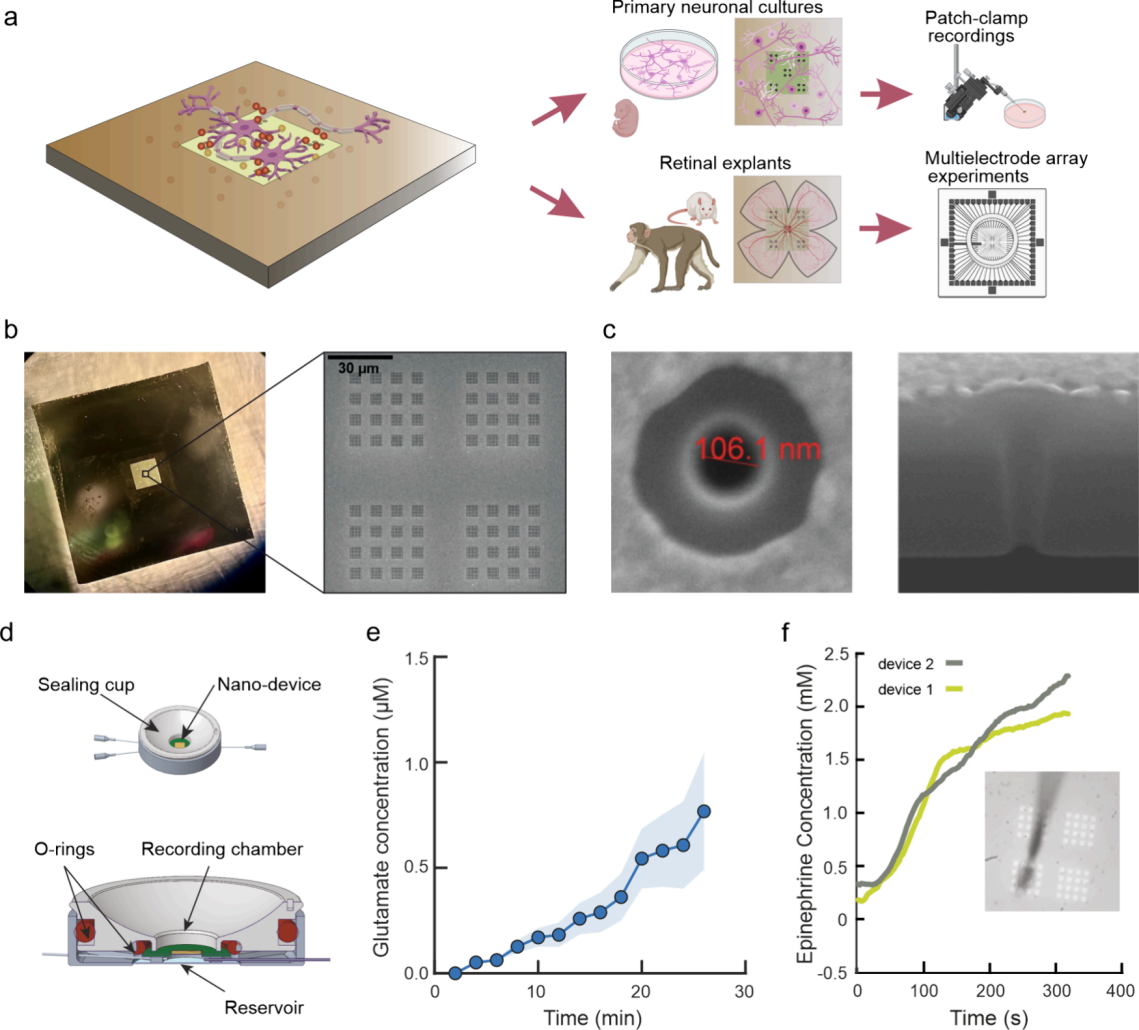
Fabrication and characterization of the nanodevice. a) Schematic representation of the nanodevice (left) and overview of tested experimental models and techniques (right). b) The top view of the nanodevice shows the transparent silicon nitride membrane and the electron microscopy detail of the nanopore pattern employed in the experiments. c) The magnification of a representative pore and its cross section present the hollow nanostructure throughout the thickness of the suspended membrane. d) Top view and section of the watertight microfluidic system used to characterize the diffusive properties of the arrays. e) Time course of glutamate diffusion through the Si_3_N_4_ nanopores array evaluated every 2 min using a bioluminescence assay upon 10 mM glutamate administration (50 μL/min) in the reservoir (mean ± SEM, *n* = 7 nanodevices). f) Time course of epinephrine diffusion through two nanodevices measured by cyclic voltammetry measurements by a CFE placed in the proximity of the nanopores (inset). The reservoir was filled with 10 mM epinephrine using a syringe pump (50 μL/min).

Electrochemical measurements gave deeper insight into the translocation dynamics through the pores. To this aim, we exploited epinephrine, a neurotransmitter belonging to the catecholamine family. Epinephrine is conventionally used for the testing of bioelectrochemical devices thanks to its electroactive groups that oxidize upon voltage application leading to quinone formation.^25,26^ Cyclic voltammetry measurements were performed by a 5 μm carbon fiber electrode positioned in the close proximity of the nanopores (Figure S2a). Once the detection of the neurotransmitter had been calibrated (Figure S2b,c), we recorded the typical oxidation current peak at 0.2 V of epinephrine diffusing through the nanopores down to the 10 mM concentration present in the reservoir. Figure 1f shows the monotonic increase of the neurotransmitter diffusing in the recording chamber as a function of time and its reproducible dynamics over different devices.

Once verified, the ability of the device to allow the passive diffusion of neurotransmitters from the reservoir to the recording chamber was verified, we investigated the feasibility to trigger a neurotransmitter-based response in excitable cells, such as cortical neurons plated on the nanopores. Before proceeding to the functional tests, we verified the biocompatibility of the devices by culturing primary cortical neurons on either nanodevices (Si_3_N_4_) or control glass coverslips (Ctrl) for 12–14 days *in vitro* (DIV) (Figure 2a). The morphology of the neuronal network, investigated both onto and outside the nanostructured surface (Figure 2b) was closely similar over the whole device. Moreover, a live fluorescence imaging assay showed that the contact with the substrates had a negligible effect on neuronal viability with comparable percentages of live cells for both Si_3_N_4_ and Ctrl surfaces (Figure S3a,b). Both experimental groups showed a similar density of neurons, immunolabeled with the neuronal marker anti-Neuronal Nuclei (NeuN), and of astrocytes, immunolabeled with the glial fibrillary acidic protein (GFAP), proving no interference of the substrate with neuronal and astroglial growth (Figure S3c,d). Finally, whole-cell patch clamp recordings demonstrated the full preservation of the passive and active properties of neurons plated on either substrate (Figure S4a). In both groups, neurons exhibited similar rheobase values and mean firing rates in response to increasing current steps (Figure S4b,c).

**Figure 2:**
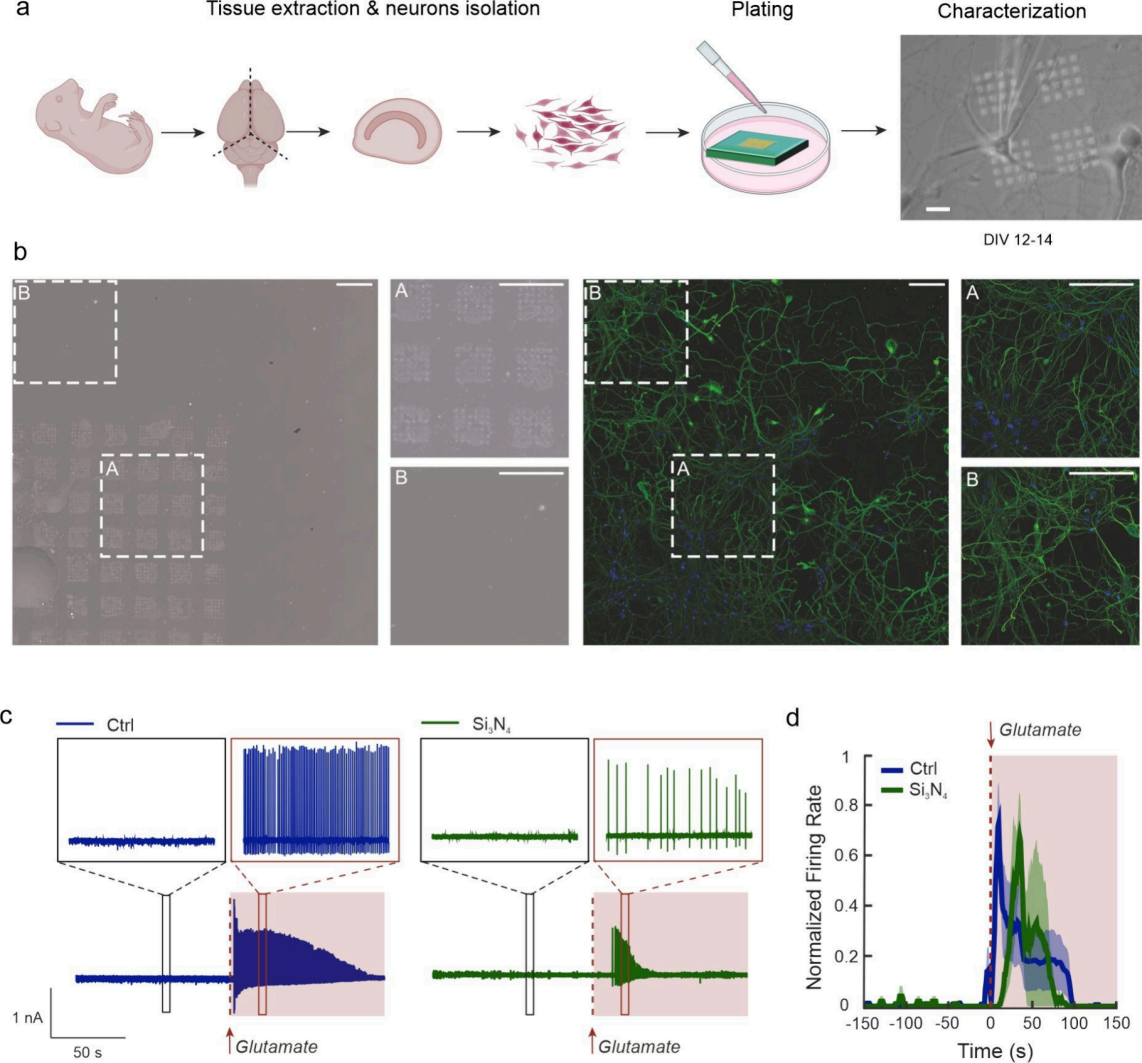
Firing of primary neurons elicited by glutamate diffusion through the nanopores. a) Schematic representation of the experiment with primary neurons. b) Representative bright field image of neurons cultured on a Si_3_N_4_ membrane showing regions with (A) and without (B) nanopores (left), and confocal fluorescence image showing neurons adhering on both areas with nuclei in blue (Hoechst) and β-III tubulin staining in green (right). The A and B areas of both bright field and fluorescence images are magnified in the lateral panels. Scale bar, 100 μm. c) Representative traces of neuronal firing activity before and after glutamate administration for both Ctrl (left, 10 mM in the pipet) and Si_3_N_4_ nanodevice (right, 10 mM in the reservoir). d) Peri-stimulus time histograms (5 s bins) of both Ctrl and Si_3_N_4_ configurations upon glutamate administration (shaded areas indicates SEM, *n* = 5, 3 neurons for Ctrl and Si_3_N_4_ respectively).

We next performed patch-clamp experiments in a cell-attached configuration on neurons cultured on Si_3_N_4_ and a reservoir glutamate concentration of 10 mM to test the stimulation capability of the devices via diffusion-based release of the excitatory neurotransmitter through the nanopores. As a control, neurons were cultured on glass coverslips and glutamate was administered by micropuffs in the proximity of the patched neuron at the same concentration used in the reservoir (10 mM for achieving a final bath concentration of 1 mM).^27−29^Figure 2c shows representative patch-clamp traces of neurons in either the Ctrl or Si_3_N_4_ configuration, revealing robust increases in the firing rate upon glutamate administration. The latency of the firing modulation evoked by glutamate was different in the two experimental groups given the intrinsic diversity of the delivery methodologies, although this confirmed the neurotransmitter diffusion dynamics from the nanodevice (10–20 s) deduced by previous investigations (Figure 2d).

Upon confirmation of the performance of the nanodevices as a promising stimulation platform for neurons, we focused on the spatial resolution of glutamate delivery from the nanopores. To this aim, we plated HEK293T cells transiently transfected with the glutamate sensor iGluSnFR3v857^30^ on the device mounted in the microfluidic system. We loaded the reservoir with 10 mM glutamate solution while acquiring time-lapse live fluorescence images of the transfected cells (Figure 3a). We considered center, surround, and periphery clusters of cells based on their distance from the nanopores (<115, 115–250 or >250 μm), as depicted in Figure 3b by purple, yellow and cyan circles, respectively, and investigated their fluorescence over time upon glutamate delivery. Figure 3c shows representative time-lapse images and the quantification of the average fluorescence intensity over time for the three populations of cells. Within a 200 s time window following glutamate administration, the cells in the central region displayed a notably greater overall increase in fluorescence with respect to those in the surrounding area. Similarly, there was a significant difference in fluorescence intensity between the surround and peripheral clusters (Figure 3d). Moreover, the higher the fluorescence intensity and therefore the glutamate concentration, the faster the response.

**Figure 3:**
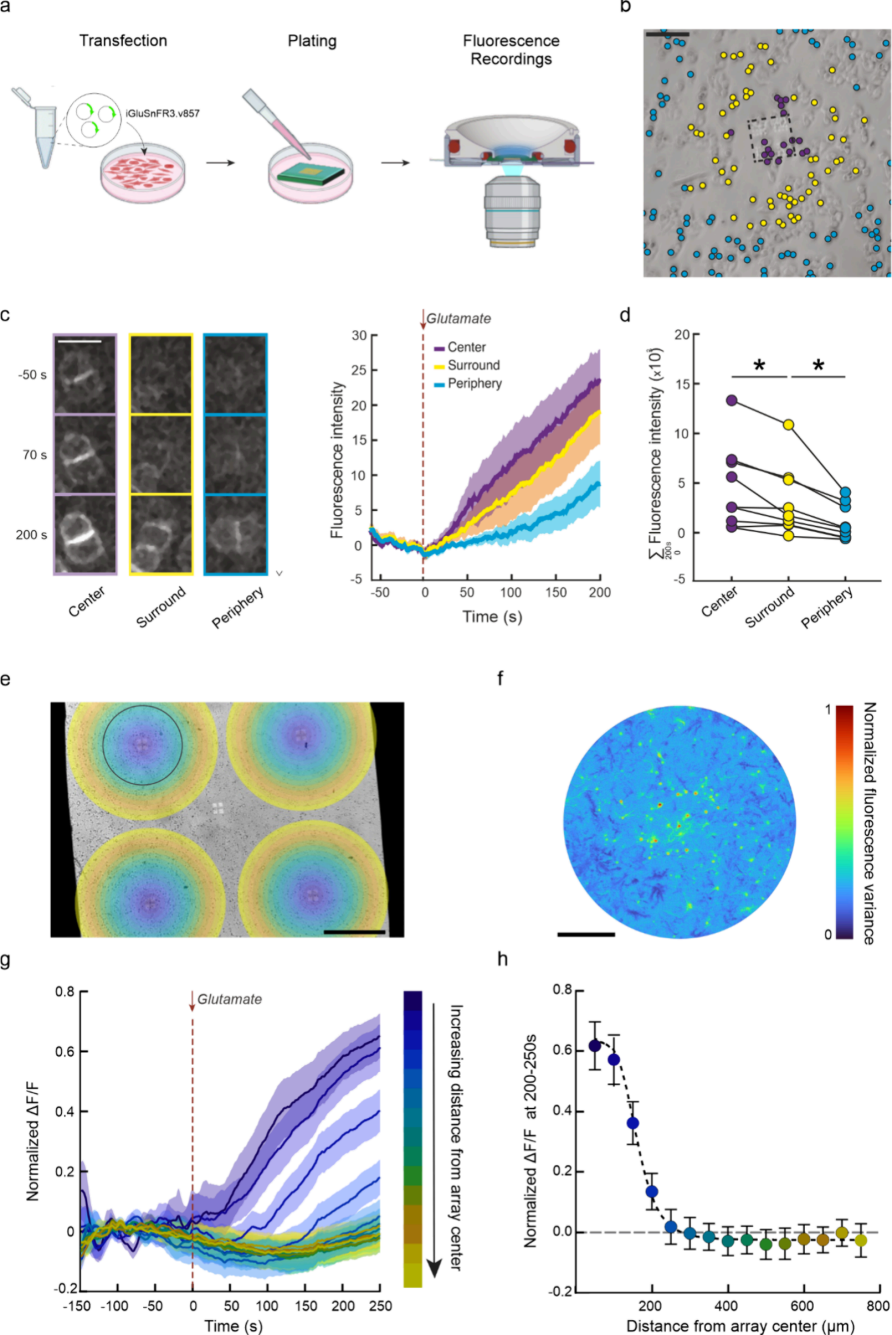
Spatially resolved glutamate diffusion through the nanodevice. a) Schematic representation of the workflow of the experiment reported in panels b–d, showing the transfection of HEK29T cells with iGluSNFR3.v857, their plating on the nanodevice and the fluorescence recordings performed 24 and 48 h after transfection, respectively. b) Representative image of HEK293T cells plated on the nanodevice highlighted in different colors based on their distance from the nanopores array (black dashed line): 0–115 μm, purple; 115–250 μm, yellow; >250 μm, cyan. Scale bar, 100 μm. c) Representative time-lapse fluorescence images (−50, 70, 200 s) of center (left), surround (middle), and peripheral (right) cells during glutamate diffusion through the nanodevice. Scale bar, 25 μm (left). Average fluorescence intensity traces of spatially sorted cells (shaded areas indicates SEM; right). d) Total fluorescence intensity after glutamate administration (*n* = 9 arrays from 2 nanodevices; *p < 0.05, one-way Anova/Holm-Šídák’s multiple comparisons tests). e) Representative bright-field image of hippocampal neurons plated on the nanodevice and labeled with the Ca^2+^ indicator Fluo-4, with superimposed circular and annular ROIs color-coded by distance from the four nanopore array centers (blue to yellow; scale bar, 500 μm). f) Normalized fluorescence variance in the proximity of the nanopore array (black circle in e) indicates higher fluorescence variation in the central region (scale bar, 200 μm). g) Average fractional fluorescence variation traces of ROIs upon glutamate administration (*t* = 0 s), from close (blue) to far (yellow) relative to the nanopore array center. Shaded areas represent SEM. h) Average fractional fluorescence values between 200 and 250 s after glutamate administration show that fluorescence intensity values steeply decrease with the distance from the nanopore array center, with a stimulation threshold at approximately 250 μm and half-maximal effect at 160 μm (sigmoidal fitting: *R*^2^ = 0.61, *n* = 12 nanopore arrays from *n* = 4 nanodevices; primary neurons from *n* = 3 independent cultures).

We then performed Ca^2+^ imaging experiments on primary neurons to assess the spatial resolution in a model of the neuronal network and physiological sensitivity to glutamate. Using the same experimental setup, we incubated neurons with Fluo-4 and acquired time-lapse live fluorescence images before and after glutamate administration in 15 concentric regions of interest (ROIs) centered on the nanopore arrays (Figure 3e). The results indicated a greater overall fluorescence increase near the center of the nanopore arrays, as depicted in Figure 3f by the representative variance map obtained from the top left array of Figure 3e. When the normalized Δ*F*/*F* is observed as a function of time, a rapid and sustained response is shown in the proximal regions, in contrast to a negligible and nearly constant fluorescence level in the distal ones (Figure 3g). To determine the stimulation spatial threshold, we regressed the normalized fluorescence values during the plateau phase (from 200 to 250 s after glutamate administration) against the distance from the center of the array (Figure 3h). The sigmoidal fitting used to characterize the fluorescence–distance relationship indicates that the semimaximal effect is reached at approximately 160 μm, with zero-effect at distances above 250 μm. These results demonstrate that glutamate can be delivered with spatial resolution, effectively targeting the location of the nanopores array.

The potential of the nanodevice as a spatially resolved neural interface for glutamate-mediated stimulation was assessed on retinal explants from rats and nonhuman primates. In the former case, we dissected the retina from albino rats and layered it on high-density multielectrode arrays (27,400 electrodes, MaxWell Biosystems) with retinal ganglion cells (RGCs) facing the electrodes and the photoreceptor side in contact with the nanopore array (Figure 4a, left). A representative activity scan of RGCs (Figure 4a, right) highlights the position of the nanopore membrane, with respect to the retinal tissue. After recording the baseline activity both below the pore array (Center) and farther from the release sites (Periphery), we evaluated the firing rate of RGCs normalized to the baseline firing frequency in the two zones upon perfusion of either glutamate (500 μM) or a control solution in the reservoir (Figure 4b,c). The glutamate-evoked activity increase over time showed a clear-cut difference between RGCs located in the proximity of the nanopores with respect to the peripheral area of the tissue, starting approximately 12–15 s after glutamate perfusion (Figure 4c). We evaluated the firing rate evolution in 5 s intervals in an early silent phase (Early) and a late plateau phase, in which spatial selectivity was relevant (Late). Explants in contact with the devices treated with physiological solution were used as controls, showing the expected uniform firing rate over time (Figure 4c and Figure S5a). The comparison of the normalized RGCs firing rate showed a significant 2-fold increase in the Late phase with respect to the Early phase, with a significantly higher firing modulation of RGCs sitting in the Center zone (Figure 4c, Figure S5a). Taken together, the *ex vivo* data confirm the *in vitro* evidence of a spatially resolved glutamatergic stimulation in an intact neural tissue

**Figure 4:**
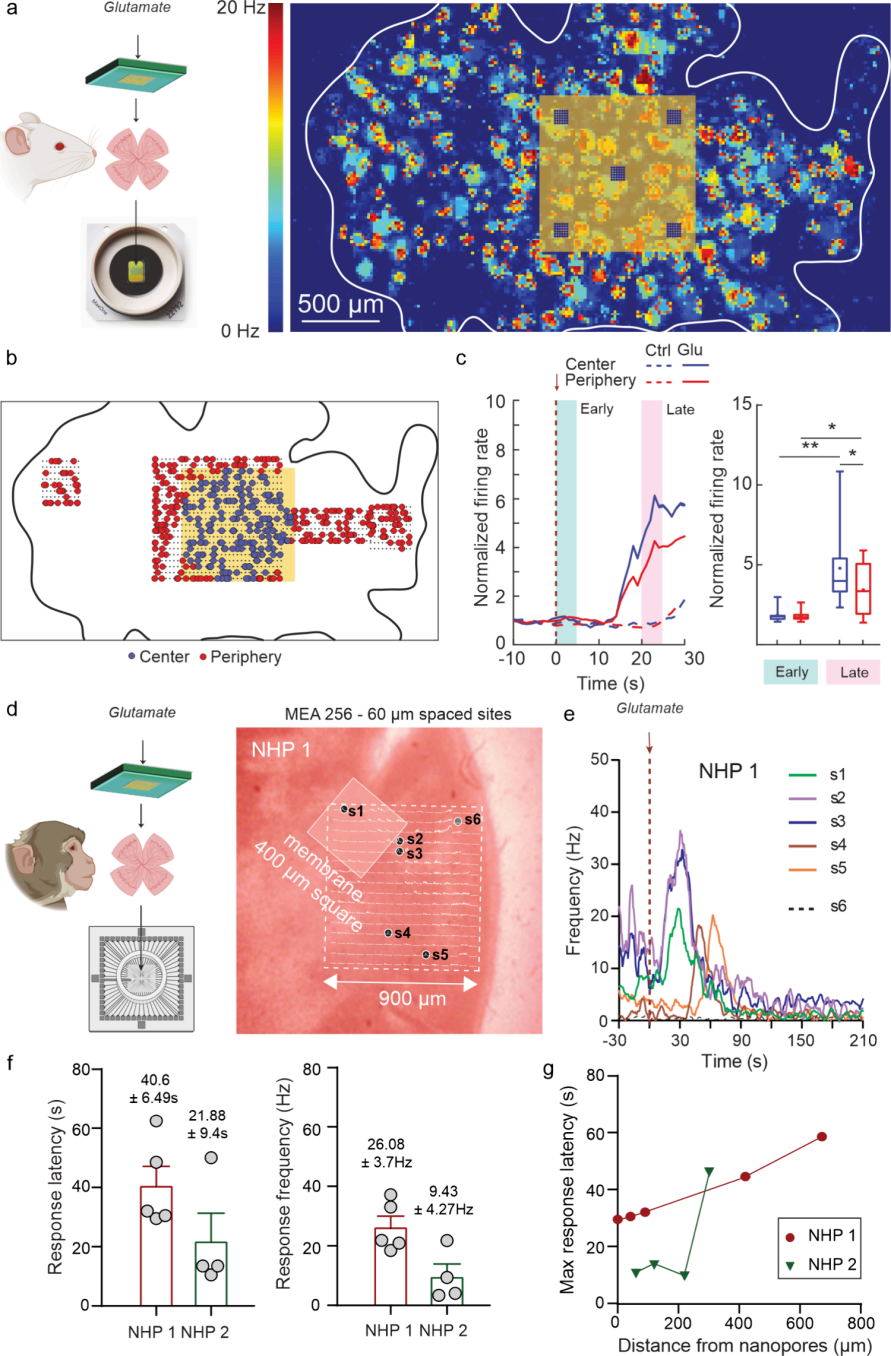
The nanodevice triggers network-mediated RGC responses in a spatially resolved manner. a) Schematic representation of the experimental setup. Retinal tissues were extracted from wild type albino rats and placed on high-density MEA platforms (MaxOne, Maxwell Biosystems, left). Representative image of the basal firing rate of a retinal explant with respect to the nanodevice position (right). b) Representative image of the sorted neuronal units clustered according to their distance from the center of the device (0–500 μm, blue; >500 μm, red). c) Representative firing rate traces normalized to baseline activity of both central (blue) and peripheral (red) clusters for either glutamate (500 μM in the reservoir, solid lines) or AMES medium (Ctrl, dashed lines) administration. *Left*: Early (0–5 s) and Late (20–25 s) time intervals after treatment are indicated with green and pink shaded areas, respectively. *Right*: Quantification of the firing rate in both Early and Late intervals (*n* = 4 animals, two-way ANOVA/Fisher’s LSD multiple comparison test, *p < 0.05, **p < 0.01). d) Schematic representation of the primate retinal explant experiment and bright field image of a retina (2–3 mm diameter disc shape) with respect to the MEA with 256 recording sites spaced by 60 μm (Multichannel Systems) and the nanodevice. e) Glutamate-triggered modulation of RGC firing frequency over time for different electrodes (S1–S5) compared to a Ctrl silent location (S6), before and during glutamate diffusion through the nanopores (50 mM in the reservoir). f) Response latency and firing frequency of two independent primate retinal explants (NHP1 and NHP2). g) Maximum response latency as a function of distance from the nanopores for both retinal explants (*n* = 2 retinal explants from 2 animals).

The spatial resolution of the proposed strategy might be better exploited in tissues with an intrinsic highly resolved spatial morphology, such as the primate retina, in which photoreceptors are small and densely packed in the fovea to provide high visual acuity. For this purpose, we prepared explants from two *post-mortem* macaque retinas (NHP 1 and NHP 2) and layered them in contact with MEAs (256 electrodes, Multichannel Systems) and our devices in the same configuration employed for rat retinal explants (Figure 4d). We identified electrodes inside and outside the nanopore membrane in both retinas (Figure 4d and Figure S5b) and analyzed the glutamate-evoked modulation of RGC firing frequency over time (Figure 4e and Figure S5b), compared to an electrode with no activity used as the noise control. We observed a difference in the latency of retinal responses at different locations. The RGC response latency in both animals was between 20 and 40 s (Figure 4f, left), with an increase of 10–30 Hz firing frequency with respect to the control electrode (Figure 4f, right) and an increase in the maximum response latency with increasing distance from the nanopores in both explants (Figure 4g). Overall, these data show the possibility to address a spatially resolved glutamatergic stimulation of neuronal networks *in vitro* and *ex vivo* that can be easily optimized and tailored for the specific application by a change in the geometry of the nanopore array and extended to other neurotransmitters or molecule delivery.

A more translational example of the proposed technology is a device restoring the activity of postsynaptic neurons that became denervated from their presynaptic partners due to neurodegeneration. To demonstrate this possibility, we tested the ability of our device in stimulating the inner retinal neurons that have lost their presynaptic inputs due to photoreceptors degeneration that, coupled with phototransduction, could open the way toward a novel prototyping of retinal prosthetic devices.^31,32^ To this aim, we used a murine model of *Retinitis pigmentosa*, the Rd1 mouse, presenting already at 30 days of age a sustained degeneration of rods later followed by cone dystrophy.^33^ Retina explants were put with inner retinal neurons in contact with the nanodevice, while the RGC activity was recorded with Ca^2+^ imaging as displayed in Figure 5a. A higher reservoir concentration (500 mM) ensured RGCs stimulation by a pressure-driven, valve-controlled perfusion system alternating with pulses of glutamate and washout. The blind retina explants exposed to 9 arrays of nanopores (Figure 5b) revealed a prompt and reproducible Ca^2+^ fluorescence increase peaking at around 60 s after each pulse (Figure 5c). The average RGC response latency, evaluated as the time corresponding to a Ca^2+^ fluorescence increase with respect to the baseline in the absence of exogenous glutamate, was approximately 6–10 s for each of the tested devices (Figure 5d). The map of the Ca^2+^-induced fluorescence increases upon a single glutamate pulse (Figure 5e and Figure S6) is representative of the average response over multiple glutamate pulses and devices (Figure 5f), showing a clear increase corresponding to the location of the nanopore arrays. The RGC response measured in four ROIs located at increasing distance from the center of the nanodevice (pink squares, Figure 5f) shows the progressive variation of the glutamate-induced Ca^2+^ signal from the center toward the edges of the membrane where there was no neurotransmitter delivery. With this nanopore array geometry, we could achieve a spatial resolution of about 70 μm upon a pulsed glutamate stimulation confirming the feasibility of the localized chemical neuromodulation.

**Figure 5:**
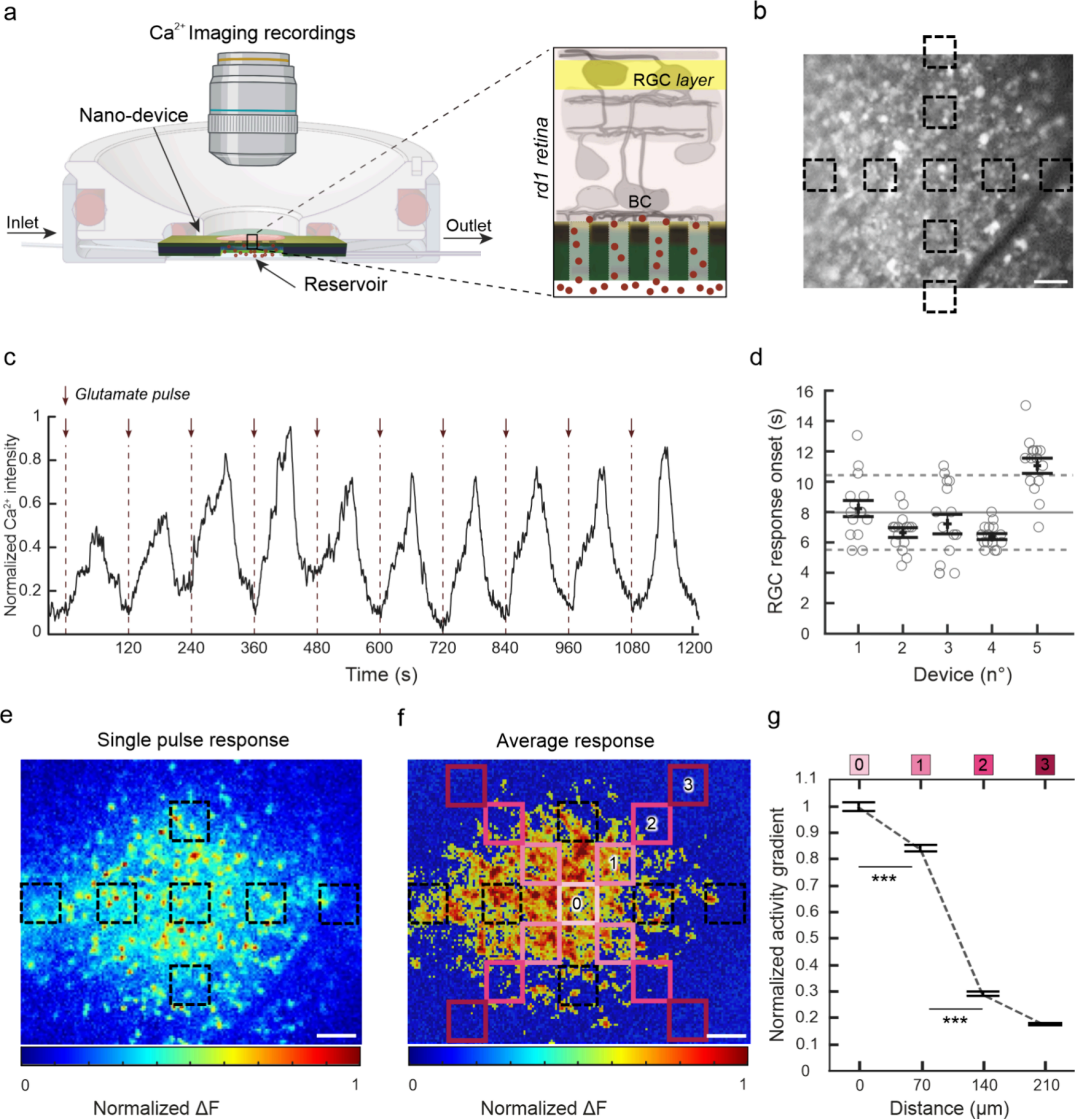
Spatial-temporal analysis of blind retinal explant responses elicited by glutamate release through the nanopores. a) Experimental setup for the testing of nanopore membranes on blind Rd1 retinal explants placed with the bipolar cells (BC) interfacing the nanopores. Glutamate (500 mM in the reservoir) was applied at 1 Hz while RGC responses were recorded by Ca^2+^ imaging. b) Fluorescence image of a retinal explant with RGCs labeled with the Ca^2+^ dye OGB-1 and the respective position of the nanopore arrays (black dashed squares). c) Representative Ca^2+^ intensity trace of glutamate-induced retinal activity. d) Estimation of average onset of RGC responses upon glutamate diffusion through the nanopores. Means ± SEM are shown together with individual experimental points. The gray solid and dashed lines represent the mean ± SD of the RGCs onset response over the 5 devices, respectively. e) Representative heatmap of the Δ*F* of the peak RGC activity upon a single glutamate pulse normalized on the min/max variation range. f) Average activity heatmap (*n* = 15 pulses, 5 devices) overlaid with ROIs with increasing distance from the center nanopore array employed for statistical analysis. g) The normalized gradient of the glutamate-mediated RGC activity shows a significant decrease from the center of the nanopore array toward the edges of the device. The activity gradient was obtained by dividing the normalized Δ*F* in each pixel by the mean normalized Δ*F* of ROI 0 (means ± SEM, *n* = 75 RGCs, 5 devices, ***p < 0.001, Mann–Whitney *U*-test. Scale bars, 50 μm.

We demonstrated that an array of nanopores realized on suspended silicon nitride membranes can be a powerful and versatile platform for a spatially resolved glutamate-mediated stimulation of primary neurons and neuronal networks, such as retinal explants from rodents and nonhuman primates. The achieved dynamics of neurotransmitter-based stimulation of cells, neurons, and tissues reflects the diffusive nature of the neural interface, while preserving the spatial resolution of the stimulation. These results demonstrate the potential of this technology that can be exploited in a variety of neurostimulation fields once properly tuned for the specific application. Indeed, different strategies can be employed to modify the latency of glutamate stimulation, such as modulation of nanopore geometry,^23^ control of neurotransmitter concentration gradient, or functionalization of the nanopore surface in terms of hydrophilicity or charge. In addition, our technology can be easily integrated with micro/nanoeletrodes^24,34^ to facilitate a micro/nanoiontophoresis (1–10 ms) that would represent a novelty among planar and multisite chemical stimulation strategies.

Overall, our results show the versatility of the solid-state nanopore system as a diagnostic platform for the chemical stimulation of complex neuronal networks and propose a promising tool for the development of hybrid neural interfaces based on neurotransmitter delivery.

## Methods

### Fabrication of Si_3_N_4_ Membranes

Polished single crystalline silicon wafers with 500 μm SiO_2_ and 500 nm Si_3_N_4_ deposited on both sides by LPCVD were purchased from University Wafer Inc. (USA). UV photolithography was employed for the fabrication of the window patterns (Shipley S1813). The exposed Si_3_N_4_ was removed by CF_4_ RIE dry etching (1000 s). A titanium adhesion layer of 5 nm and a conductive gold layer (20 nm) were evaporated on the membrane top side. Nanopores of 100 nm were patterned on the top of the Si_3_N_4_ membranes by Focused Ion Beam (FIB) milling, with a FEI Helios Dual Beam. Nanopore arrays underwent oxygen plasma ashing on both sides to increase the hydrophilicity of the surface and then sterilized for 2 h at 120 °C in a dry oven to guarantee full wettability and sterility.

### Cyclic Voltammetry

Manually cut carbon fiber electrodes of 5 μm in diameter (CFE-2, ALA Scientific Instruments) were connected to a HEKA EPC10 USB Amplifier and placed in the proximity of the nanopores to detect epinephrine. Scan rates were maintained between 0.1 and 1 V/s. Voltage application and current recordings were handled with PatchMaster (HEKA Elektronik). For further details, see the Supporting Information.

### Primary Neuron Preparation

Primary neurons were obtained as previously described.^35^ Briefly, WT C57bl/6 J (Charles River) mice were sacrificed by CO_2_ inhalation and cervical dislocation, and 17/18-days embryos were promptly removed by cesarean section. Brain cortices were dissected and incubated in 0.125% trypsin for 20 min at 37 °C for enzymatic digestion. Cells were then mechanically dissociated with a fire-polished Pasteur pipet, and cell viability and number was determined by Trypan Blue exclusion assay. Neurons were plated on poly-l-lysine-coated Si_3_N_4_ membrane or glass coverslip in Neurobasal medium containing 2% B27, 1% Glutamax and 1% penicillin/streptomycin. The nanopore arrays were placed on polymeric elevated supports (Sylgard 184, Corming) to enable medium diffusion through the nanostructures. The samples were incubated for 12–14 DIV prior to the experiments.

### Imaging and Immunocytochemistry

Neurons were fixed in 4% paraformaldehyde for 15 min at room temperature and washed with PBS. Samples were then incubated overnight with primary mouse monoclonal anti-β-tubulin III (#801202, BioLegend), guinea-pig antiastrocyte-specific type-III intermediate filament protein (GFAP, #173004, Synaptic-Systems), and rabbit anti-Neuronal Nuclei (NeuN, #12943, Cell Signaling) diluted in 5% NGS (normal goat serum)/0.1% Triton/PBS. Samples were incubated for 2 h with secondary antibody (Alexa-568 goat antirabbit #11036, Alexa-647 goat antiguinea pig #21450; Alexa-488 antimouse #A11029, Thermo-Fisher Scientific), and finally with Hoechst 33342 for 5 min before being mounted with Vectashield medium (#H-1000–10, Vector Laboratories). For further details, see the Supporting Information.

### Patch-Clamp Recordings

All recordings were performed using a HEKA EPC10 USB amplifier (HEKA Elektronik) as previously described.^35^ During recordings, cells were maintained in extracellular Tyrode solution containing (in mM) 140 NaCl, 2 CaCl_2_, 1 MgCl_2_, 4 KCl, 10 glucose, and 10 HEPES (pH 7.3 with NaOH). The internal solution was (in mM): 126 K Gluconate, 4 NaCl, 1 MgSO_4_, 0.02 CaCl_2_, 0.1 BAPTA, 15 Glucose, 5 HEPES, 3 ATP, and 0.1 GTP (pH 7.2 with KOH). Action potentials were recorded by clamping at a holding potential 0 mV in “on-cell” mode for cell-attach configuration, while a holding potential of −70 mV was employed for whole-cell configuration. Data were acquired at 20 kHz and highpass filtered. Firing rate traces were obtained by integrating the spike count in 5 s bins. Neurons plated on the nanopore arrays underwent automatic glutamate administration via an external syringe pump (50 μL/min, 4 min, Harvard Apparatus), while neurons plated on control coverslips were cued with manual glutamate administration at 1 mM in the bath.

### Time-Lapse Fluorescence Recording

HEK293T cells were grown in DMEM-F12 supplemented with 0.5 mM pyruvate, 10% fetal bovine serum, 1% l-glutamine, and 1% penicillin/streptomycin. HEK293T cells were transfected using 2 μL of Lipofectamine 2000 (Invitrogen) according to the manufacturer’s protocol (iGluSnFR3.v857.SGZ #178334 Addgene). Transfected HEK-293T were plated on the devices and the day after evaluated by time-lapse fluorescence imaging. Fluorescence videos were recorded for 260 s (60 s baseline, 200 s after glutamate administration) with an ORCA-R2 C10600-10B instrument (Hamamatsu Photonics, 500 ms exposure). For further details, see the Supporting Information.

### Retina Explants Electrophysiology and Calcium Imaging

Retinas were dissected from albino wild type rats and *Macaca fascicularis* and Rd1 blind mice and kept in oxygenated (5% CO_2_, 95% O_2_) extracellular medium. The activity of RGCs was recorded by high- or low-density MEAs (MaxOne, Maxwell Biosystems and MEA 256 chip, Multichannel systems) and by calcium imaging when layering the nanopores on the subretinal side of the explants and upon glutamate diffusion. For further details, see the Supporting Information.

## Supporting Information Available

Nanodevice fabrication, SEM images, CV, cell viability, electrophysiological properties, firing activity, heatmaps, and methods, (PDF)
