# Supplementary material for: Solid-State Nanopores for Spatially Resolved Chemical Neuromodulation
Source: Nano Lett. 2024 Nov 19;24(48):15215–25. doi: 10.1021/acs.nanolett.4c02604 (PMC11622382; doi:10.1021/acs.nanolett.4c02604)
Supplement: Supplementary file 1 — nl4c02604_si_002.pdf [file nl4c02604_si_002.pdf]

Solid state nanopores for spatially resolved chemical neuromodulation

F. Vacca[1,2§], F. Galluzzi[1,3§], M. Blanco-Formoso[4,5§], T. Gianiorio[1,6], Angela F. De Fazio[4], F. Tantussi[4], S. Stürmer[7], W. Haq[7], E. Zrenner[7], A. Chaffiol[8], C. Joffrois[8], S. Picaud[8], F. Benfenati[1,2], F. De Angelis[4#], E. Colombo[1,#§]*

[1] Center for Synaptic Neuroscience and Technology, Istituto Italiano di Tecnologia, 16132, Genova, Italy

[2] IRCCS Ospedale Policlinico San Martino, 16132, Genova, Italy

[3] The Open University Affiliated Research Centre at Istituto Italiano di Tecnologia (ARC@IIT), Genova, Italy

[4] Plasmon Nanotechnology, Istituto Italiano di Tecnologia, 16163, Genova, Italy

[5] CINBIO Universidade de Vigo, 36310, Vigo, Spain

[6] Department of Neuroscience (DINOGMI) University of Genoa, 16132, Genova, Italy

[7] Centre for Ophthalmology, Institute for Ophthalmic Research, University of Tübingen, 72076, Germany

[8] Institut de la Vision, Sorbonne Université, 75012, Paris, France

[§] *F.V., F.G., and M.B.F. contributed equally to this work*

[#] *E.C. and F.D.A. contributed equally to this work*

[*] Email: elisabetta.colombo@iit.it

SUPPLEMENTARY FIGURES

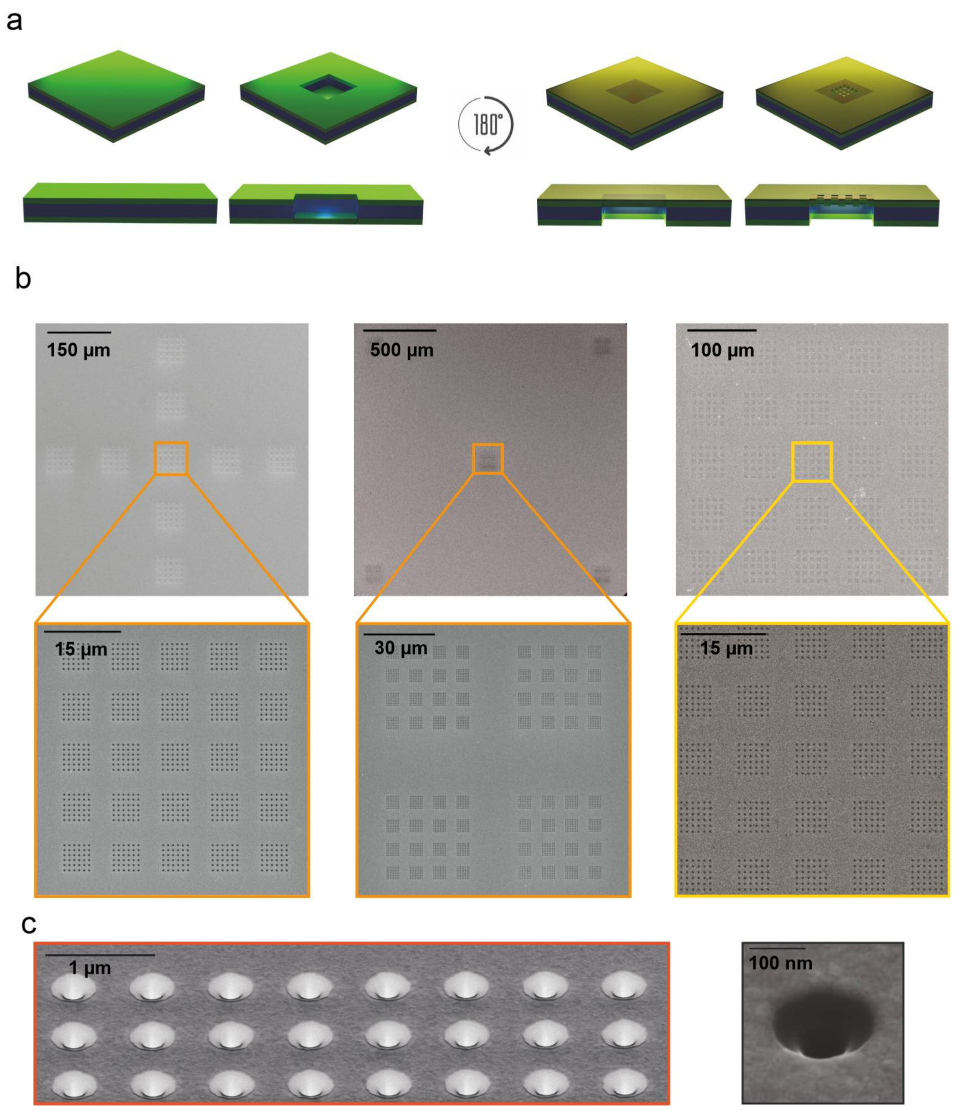

Supplementary Figure 1. Nanodevice fabrication and SEM characterization. a) Schematic representation of the nanodevice, bottom view (left) and top view (right). b) SEM images of the different nanopore array geometries used for the following experiments: Rd1 retinal explants, Ca^{2+} imaging (left); glutamate bioluminescence assay, cyclic voltammetry, electrophysiology on primary neurons/rat retina explants, Ca^{2+} imaging in HEK293 cells and neurons (middle); MEA recordings on non-human primate retina explants (right). c) Representative SEM micrograph showing the reproducibility of milling.

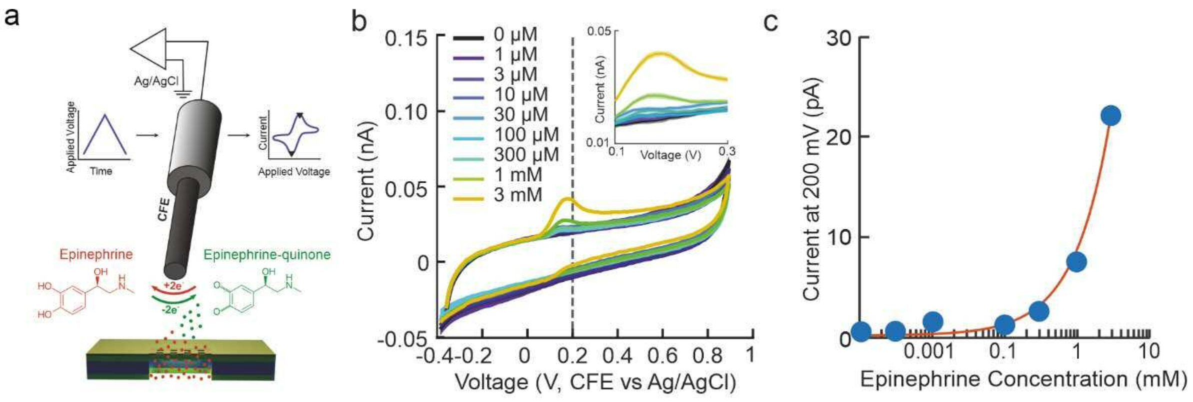

Supplementary Figure 2. Cyclic voltammetry measurements. a) Schematic representation of the electrochemical experiment performed to characterize epinephrine diffusion from the nanopore array. A voltage triangular wave was used to polarize the carbon fiber electrode (CFE-2) placed on top of the nanopores. b) Characterization of the epinephrine CFE-2 electrode sensitivity. Cyclic voltammetry protocols were performed to record the resulting current in the presence of increasing concentrations of epinephrine (1-3 mM). Inset shows magnification around +200 mV (shaded areas indicate SD for n = 50 cycles for each concentration). c) Linear regression of epinephrine concentration vs. current values at + 200 mV.

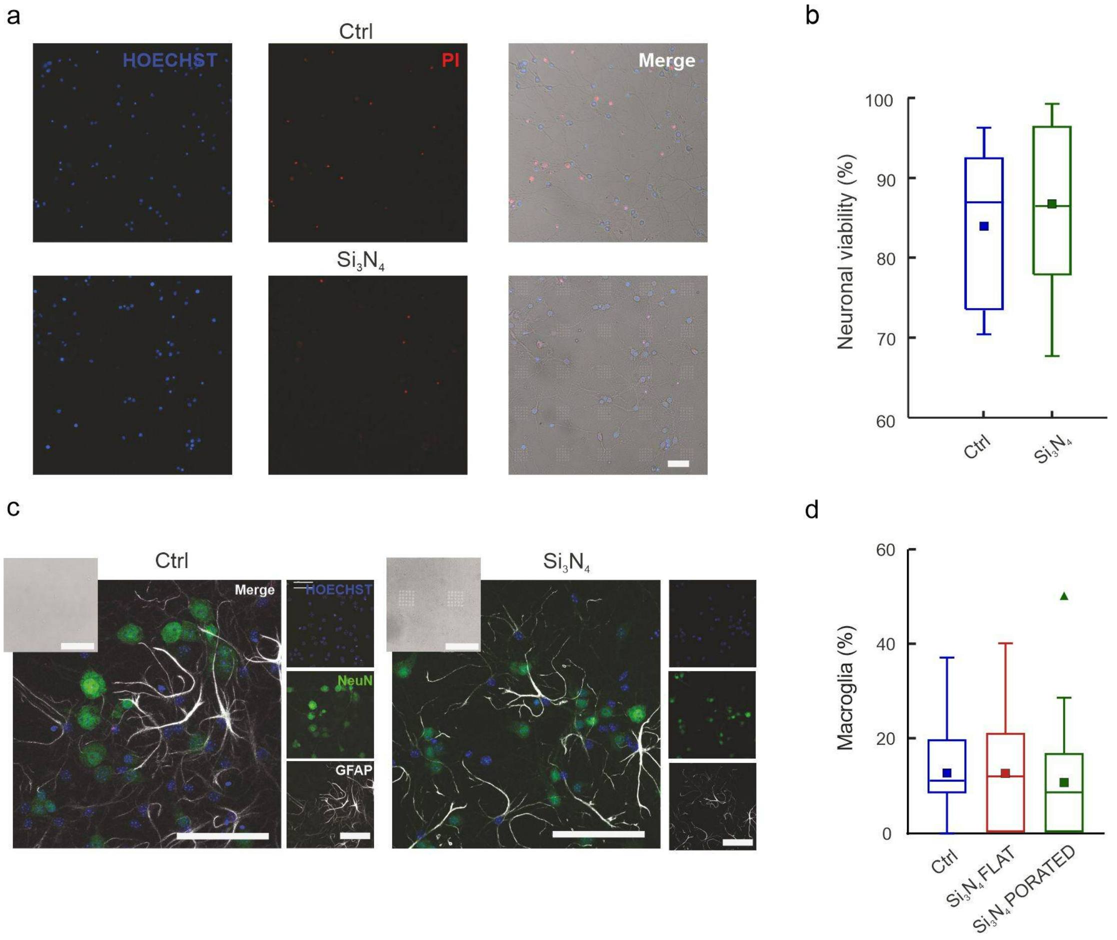

Supplementary Figure 3. Preservation of viability of primary neurons grown onto the nanodevices. a) Representative images of cell viability in Ctrl coverslips and Si_3N_4 membranes showing Hoechst-positive cell nuclei (blue; total cells), propidium iodide-stained cells (red; dead cells), and the respective bright field images. b) Quantification of cell viability in both experimental groups (n = 9 for both Ctrl and Si_3N_4 from 3 independent cultures). c) Representative images of primary neurons plated on Ctrl and Si_3N_4, labeled with Hoechst (cell nuclei, blue) and immunostained with neuronal (NeuN, green) and astroglial (GFAP, white) markers. d) Quantification of the percentage of astrocytes shows the absence of a significant astrogliosis induced by the contact with the device. (n = 5 for both Ctrl and Si_3N_4 from 5 independent cultures). Scale bar, 100 µm.

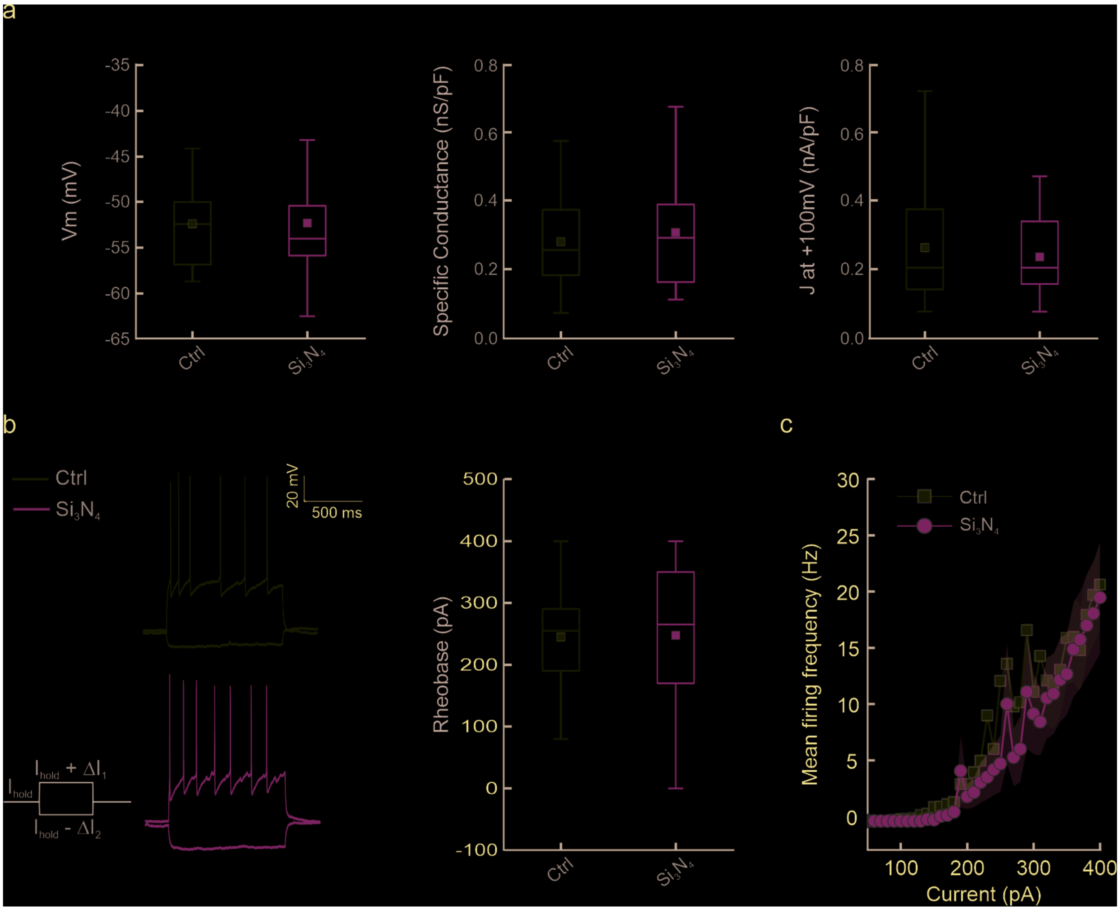

Supplementary Figure 4. Preservation of the electrophysiological properties of neurons grown onto the nanodevices. a) The analysis of the passive properties of patched neurons grown on either Ctrl or Si_3N_4 shows that both substrates are comparable in terms of resting potential (left), specific conductance (middle), and current density (right). b) Representative traces of current-clamped firing activity of the neurons for both experimental groups. Rheobase currents are comparable across substrates. c) Mean firing frequency in response to increasing injected currents is unaltered by the nanodevices with respect to control (n = 22 and 18 neurons for Ctrl and Si_3N_4 respectively, from 3 independent culture preparations).

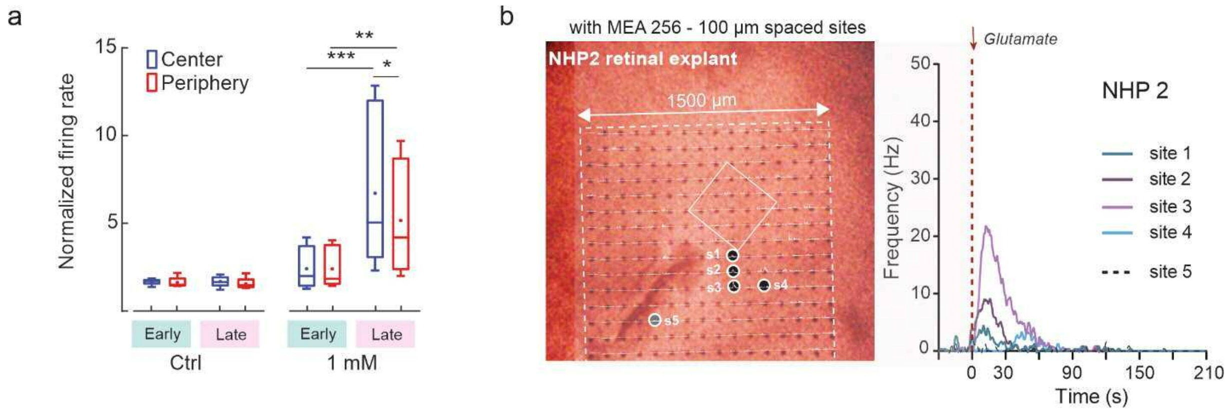

Supplementary Figure 5. Glutamate-induced firing activity in the rat and macaque retina. a) Comparison of the normalized firing rate during Early and Late intervals for 0 and 1 mM glutamate concentrations in the reservoir (n = 4 animals, two-way ANOVA/Fisher's LSD multiple comparison tests; *p<0.05, **p<0.01, ***p<0.001. b) *Left:* Representative image of a non-human primate (NHP2) retina explant superimposed with MEA 256 and nanodevice layouts with superimposed traces of their activity at different sites from the nanopores array (S1-S4) with respect to a silent control electrode (S5). *Right:* Glutamate-triggered RGCs firing activity over time for the different active sites.

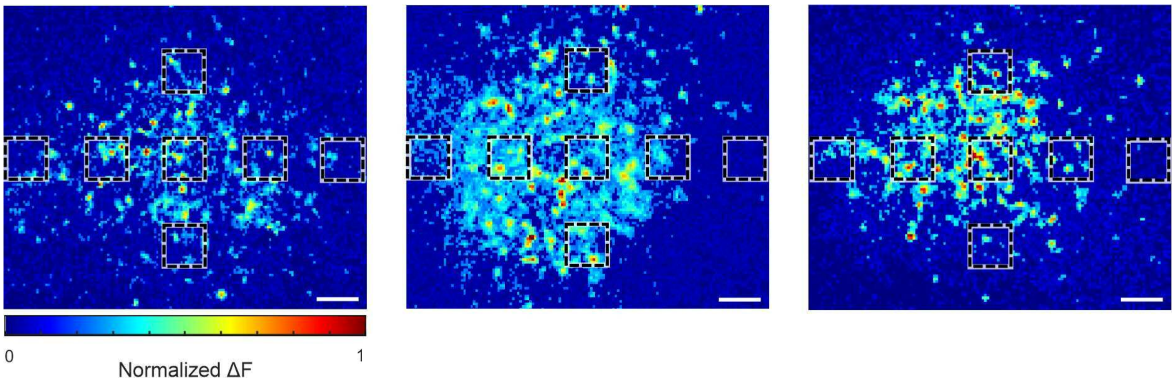

Supplementary Figure 6. Representative Ca^{2+} signal heatmaps in Rd1 retinal explants evoked by a single glutamate pulse in distinct nanodevices. Representative heatmaps of the ΔF of the peak RGC activity upon a single glutamate pulse normalized on the min/max variation range (see pseudo-color table below) overlaid with the nanopore arrays locations. Scalebars, 50 µm.

SUPPLEMENTARY METHODS

Bioluminescence glutamate assay. Glutamate diffusion through the nanopore device was evaluated by a Glutamate-Glo Assay (Promega) that exploits a luciferase-based sensor to provide a selective measurement of glutamate concentration upon proper calibration. The nanodevices were mounted onto the microfluidic chamber shown in Figure 1d. The recording chamber was filled with 300 µl of physiological solution, while the reservoir was filled with a syringe pump at 50 µl/min with 10 mM glutamate solution. The glutamate translocation was measured every 2 minutes by collecting 50 µl from the recording chamber and immediately refilled with 50 µl physiological solution. The biolumenscence recordings were performed simultaneously by a plate-reader recording (Tecan Spark).

Cyclic voltammetry. CFE-2 were manually cut and their active area was calculated running cyclic voltammetry protocols (voltage ramp, -0.3-0.6 V; scan rate, 100 mV/s) in 1 mM ferricyanide/0.5 M KCl solution (pH 3.0)[1]. To link oxidative currents and epinephrine (Sigma-Aldrich, E4642) concentration we ran cyclic voltammetry protocols (voltage ramp, -0.4-0.9 V; scan rate, 1 V/s, 100 repetitions) at different concentrations (10, 30, 100, 300, 1000, 3000 µM) of epinephrine. We subtracted the baseline current in PBS solution to the current for each epinephrine concentration, and we obtained the calibration curve from the linear regression of the current values at 200 mV for each epinephrine concentration. We loaded by an external syringe pump (Harvard Apparatus, velocity 50 µl/min), 10 mM epinephrine in the reservoir of the fluidic system encasing the nanodevice. The CFE-2 electrode was placed in the close proximity of the nanopores and voltage ramps of -0.4-0.9 V and a scan rate of 1 V/s were applied to measure the oxidative currents elicited by the epinephrine translocated through the nanopores by diffusion. We ran cyclic voltammetry for a total of 500 s (180 s before and 320 s after epinephrine administration from the reservoir).

The epinephrine concentration traces as a function of time were finally smoothed by a moving average filter with 30 s span.

Live imaging and analysis of SF-Venus-iGluSnFR. For the live fluorescence assays to quantify primary cortical neurons viability on the nanopore arrays and compare them to control preparations, live neurons at 12 DIV were stained with propidium iodide (PI, 1 μM) and Hoechst 33342 (Hoechst, 1 μM) for 3 min. PI, not permeant to live cells, was used to quantify cell death and Hoechst to visualize nuclei. The quantification of the viability was obtained by the ratio of PI-positive to Hoechst-positive cells.

The live fluorescence assay in iGluSnFR3v857-transfected HEK-293T was used to assess the spatial selectivity of the glutamate diffusion through the nanodevices. Glutamate sensitive cells were selected upon manual segmentation depending on the standard deviation of their fluorescence intensity over time. All fluorescence values were normalized to the average baseline fluorescence. Bright field images were used to map the position of nanopores and sort the segmented cells depending on the distance from the center of the nanopores (<115 μm; 115-250 μm; >250 μm). Ultimately, time-fluorescence traces were smoothed using a moving average filter with 2.5-s span.

Primary neurons calcium imaging. Primary hippocampal neurons, obtained as described previously, were cultured on nanodevices. At 12 days in vitro (DIV), the neurons were incubated with 0.5 μM Fluo-4 (ThermoFisher Scientific, F14201) for 30 min, rinsed and returned to the incubator for 10 min before imaging. During recordings (Nikon Eclipse Ni, 4x magnification, FITC filter), cells were maintained in extracellular Tyrode's solution with the following composition (in mM): 140 NaCl, 2 $CaCl_2$, 1 $MgCl_2$, 4 KCl, 10 glucose, and 10 HEPES (pH 7.3, adjusted with NaOH). As done in patch-clamp recordings, neurons plated on nanopore arrays

underwent automatic glutamate administration via an external syringe pump (50 µl/min for 4 min, Harvard Apparatus). Calcium imaging analysis was performed as follows: we manually selected the center of the four corner nanopore arrays and defined 15 concentric regions of interest (ROIs) starting from the center of each nanopore array. The first circular ROI had a radius of 50 µm, followed by annular ROIs with increasing radius (100-750 µm, 50 µm steps). For each ROI, the mean fluorescence was measured before, during, and after glutamate administration and normalized to the average baseline fluorescence (F_0), calculated over the pre-stimulation period (from t = -150 s to t = -30 s). The ΔF/F was then calculated as ΔF=F(t)/F0-1. To better distinguish the fluorescence variations based on distance from the nanopore arrays, we applied a secondary normalization across all ROIs within the same nanopore array, relative to the maximum fluorescence observed during the entire recording. To estimate the threshold distance for stimulation, we analyzed the average normalized fluorescence change during the late glutamate stimulation phase (200 to 250 s after administration) as a function of distance from the nanopore array center. A sigmoidal curve fit was applied to this data to determine the threshold distance for stimulation.

Ethical approval and animal handling. All animal procedures were performed in accordance with the National Institutes of Health Guidelines (1996) and the guidelines established by the European Community Council (Directive 2012/63/EU of 22 September 2010) and respective National regulations and authorizations. The study was performed in accordance with the ARVO statement for the use of animals in ophthalmic and visual research and approved by the Tübingen University Committee on Animal Welfare (Einrichtung für Tierschutz, Tierärztlichen Dienst und Labortierkunde), the Animal Welfare/Ethics Committee (Organismo Preposto al Benessere Animale, OPBA) of the animal facility of the Ospedale Policlinico San Martino, and Animals

housing conditions, surgical procedures and experimental protocols were performed in strict accordance and after validation of the European Council Directive (2010/63/EU). All procedures were compliant with §4 of the German law on animal welfare (permission for experiments on retinal tissue after sacrifice; protocol code: Mitteilung nach § 4 Abs. 3 TierSchG and approval: AK 04/21M) and the Italian authorization n. 24237 24/06/2021.

Rat retina explants. Rats were euthanized using CO_2 inhalation followed by cervical dislocation. Eyes were then removed, and eyecups were placed in AMES medium (CASA) bubbled with 5 % CO_2 and 95 % O_2. Retinas were carefully dissected from the sclera in bubbling AMES medium. Neural activity of rat RGCs was recorded using a MaxOne system[2] (Maxwell Biosystems), selecting up to 1024 electrodes for simultaneous recordings. For each experiment, a piece of isolated retina was placed RGC-side down in a recording chamber previously coated with 0.01 % Poly-DL-ornithine (Merck, #P8638). Electrode contact was ensured by gently applying pressure on the photoreceptor side of the retina using a transparent permeable membrane and 5 % CO_2 and 95 % O_2 bubbling was supplied directly into the chamber. Extracellular activity was recorded at 20 kHz using MaxLab software. Following an assay provided by the MaxLab software to obtain electrical footprints of spontaneous activity, a custom-made tissue holder embedding the prototype was introduced into the chamber. A green light LED beam was focused through the tissue holder and the device to elicit light artefacts selectively beneath the device. Guided by light artefacts, the device was positioned over the active region of the retinal explant, and electrode configuration was adjusted to cover both the device area and a peripheral region. Glutamate was administered using a remotely controlled micro-valve system, controlled by TTL signals generated through MATLAB, Version R2022b (The MathWorks, Inc) and an Arduino UNO platform. We performed spike sorting by an offline automatic algorithm[3] and manually curated results with a custom-made

MATLAB application judging the quality of all sorted units depending on inter-spike intervals and spiking amplitude coherence. Only units with firing rate greater than 1 Hz were considered. The remaining units were then spatially divided depending on the distance from the center of the devices. For each neural unit, we calculated the ratio of the average firing rate during two time windows following glutamate administration (0-5 seconds and 20-25 seconds) to the average firing rate during the 5 seconds immediately preceding glutamate administration. The overall activity of the two regions, for each retinal explant, was then calculated as the average ratios of the populations.

Rd1 retina explants. We used a blind rd1 (C3H Pde6brd1/rd1) mouse model (age: 19–30 days; n = 5) for retinal degeneration with primary rod degeneration and secondary cone dystrophy[4]. The animals were housed under standard light conditions, had free access to food and water, and were used irrespective of gender. The animals were anesthetized in a carbon dioxide atmosphere and were immediately sacrificed by cervical dislocation. After enucleation of the eyes, retinas were isolated in artificial cerebrospinal fluid (ACSF) containing (in mM): 125 NaCl, 26 $NaHCO_3$, 2.5 KCl, 2 $CaCl_2$, 1 $MgCl_2$, 1.25 NaH_2PO_4 and 20 glucose. The pH was kept at 7.4 by carboxygen perfusion (95 % CO_2/5 % O_2). RGCs were bulk-loaded with the fluorescent Ca^{2+} indicator Oregon Green 488 BAPTA-1 (OGB- 1) by electroporation as previously described[5].

The nanodevice was integrated into the custom microfluidic system for the glutamatergic stimulation of the retinal explants placed on the membrane of the nanodevice with the outer retina interfacing the nanopores. The inlets of the microfluidic system were connected to the corresponding reservoirs of glutamate (500 mM, L-Glutamic Acid) and ACSF washout solutions by a pressure-driven, valve-controlled perfusion system (valve manifold: ALA-VM8, valve

control: VC3, air pressure: PR-10, ALA Scientific Instruments). All chemicals were obtained from Sigma-Aldrich.

The RGCs activity was recorded performing Ca^{2+} imaging recordings using an upright fluorescence microscope (BX50WI, Olympus) with a 20 X water immersion objective. The imaging system is equipped with a polychromator (VisiChrome, Visitron Systems, Puchheim, Germany) and a CCD camera (RETIGA-R1, 1360 × 1024 pixels, 16 bit). Image stacks of the OGB-1 fluorescence were acquired at 2 Hz (470 nm excitation; Olympus U-MNU filter set, 20 ms exposure time, 8-pixel binning) using VisiView software (V 3.1, Visitron Systems). The pulsed glutamate stimulation was synchronized with the Ca^{2+} acquisition and controlled by a TTL from a stimulus generator (STG2008, MultiChannel Systems MCS GmbH).

ImageJ and MATLAB were employed for the analysis of the Ca^{2+} fluorescence intensity traces and maps. Once ROIs were manually chosen on RGCs soma in the proximity of the nanopores, the glutamate-induced RGCs activity onset was evaluated as the time interval between the glutamate pulse start time and the moment when the Ca^{2+} fluorescence intensity increased above a baseline threshold. We considered for each pulse a lag time of 500 ms to fill the reservoir after washout. Alternatively, to estimate the RGCs activity modulation as a function of the distance from the nanopores, we realized fluorescence heatmaps considering the peak fluorescence intensity for each glutamate pulse and analysed the average OGB-1 intensity in square ROIs with different distance from the center of the array.

Primate retinal explants. Experiments were performed on 2 non-human-primates (*Macaca fascicularis*). MEA recordings were conducted using retinal peri-foveal explants (without the retinal pigment epithelium attached). First, explants (~ 2 mm diameter) after 24 h of incubation in tissue culture medium were positioned on a cellulose membrane and delicately pressed against a

MEA chip (MEA256 60/10 iR-ITO or 100/30 iR-ITO; MultiChannel Systems MCS GmbH) using a micro-manipulator and a descender, with the RGCs facing the electrodes.

The retina was continuously perfused with Ames medium (Sigma-Aldrich) bubbled with 95 % O_2 and 5 % CO_2 at 34°C fluxed at a rate of 2-3 ml/min during experiments. Action potentials were identified on the filtered electrode signal (second-order high-pass Butterworth, cutoff frequency 200 Hz), with a threshold of at least 4 times the SD of the signal. The retinas were dark-adapted for 20 min in the recording chamber before recordings. Glutamate was diluted in AMES medium to a final concentration in the reservoir of 50 mM.

Statistical analysis. Box plots show the 25th to 75th percentiles (box), median (line), mean (square), whiskers length refers to min-to max values and outliers are handled with Tukey method. All statistical tests were ran using GraphPad Prism 10. The number of samples necessary for the experiments was preliminarily calculated based on the experimental variability and need to reach an appropriate number of replications for a robust statistical analysis. The number of animals/independent cell preparations for the planned experiments (sample size, n) was predetermined using the G*Power software considering values of $\alpha = 0.05$ and at least 80% power. Data are expressed as means ± SEM unless specified otherwise for number of independent animals/cell preparations (n) with superimposition of the individual experimental points or as box plots (center line, median (Q2); square, mean; box limits, 25th (Q1)-75th (Q3) percentiles; whisker length refers to min-to max values). Normal distribution was assessed using the D'Agostino–Pearson normality test and accordingly parametric or non-parametric statistical tests have been employed.

REFERENCES

(1) Schulte, A.; Chow, R. H. A Simple for Insulating Microelectrodes Using Anodic Electrophoretic Deposition of Paint. *Anal Chem* **1996**, *68* (17), 3054–3058. https://doi.org/10.1021/AC960210N/ASSET/IMAGES/MEDIUM/AC960210NE00003.GIF.

(2) Müller, J.; Ballini, M.; Livi, P.; Chen, Y.; Radivojevic, M.; Shadmani, A.; Viswam, V.; Jones, I. L.; Fiscella, M.; Diggelmann, R.; Stettler, A.; Frey, U.; Bakkum, D. J.; Hierlemann, A. High-Resolution CMOS MEA Platform to Study Neurons at Subcellular, Cellular, and Network Levels. *Lab Chip* **2015**, *15* (13), 2767–2780. https://doi.org/10.1039/C5LC00133A.

(3) Buccino, A. P.; Hurwitz, C. L.; Garcia, S.; Magland, J.; Siegle, J. H.; Hurwitz, R.; Hennig, M. H. SpikeInterface, a Unified Framework for Spike Sorting. *Elife* **2020**, *9*, 1–24. https://doi.org/10.7554/ELIFE.61834.

(4) Keeler, C. E. The Inheritance of a Retinal Abnormality in White Mice. *Proc Natl Acad Sci U S A* **1924**, *10* (7), 329–333. https://doi.org/10.1073/PNAS.10.7.329.

(5) Peiroten, L.; Zrenner, E.; Haq, W. Artificial Vision: The High-Frequency Electrical Stimulation of the Blind Mouse Retina Decay Spike Generation and Electrogenically Clamped Intracellular Ca2+ at Elevated Levels. *Bioengineering* **2023**, *10* (10), 1208. https://doi.org/10.3390/BIOENGINEERING10101208/S1.
